# Efficacy of transanal drainage tubes in postoperative anastomotic leakage in patients with laparoscopic anterior rectal resection without diverting stoma

**DOI:** 10.1038/s41598-025-03440-7

**Published:** 2025-05-29

**Authors:** Guancong Wang, Haiwen Tang, Ying Huang, Yincong Guo

**Affiliations:** 1 Department of Colorectal Surgery, Zhangzhou Affiliated Hospital of Fujian Medical University, Zhangzhou, 363000 China; 2 Department of Colorectal and Anal Surgery, Zhangzhou Municipal Hospital of Fujian Province, Zhangzhou, 363000 China; 3 Department of General Surgery, Zhangzhou Affiliated Hospital of Fujian Medical University, Zhangzhou, 363000 China; 4 Department of Colorectal Surgery, Fujian Medical University Union Hospital, Fuzhou, 350001 China; 5 Department of Colorectal and Anal Surgery, Zhangzhou Affiliated Hospital of Fujian Medical University, Zhangzhou, 363000 China

**Keywords:** Transanal drainage tube, Anastomotic leakage, Rectal cancer, Propensity-score matched, Gastroenterology, Oncology

## Abstract

To assess whether transanal drainage tubes (TDT) protect against anastomotic leakage (AL) in patients without diverting stomas (DS) after laparoscopic anterior rectal resection (LAR). In the TDT group, after anastomosis of the sigmoid colon to the rectum, a 32F silicone tube was inserted through the anus with the tip of the tube exceeding the anastomosis by more than 5 cm. The tube was secured around the anus with a skin suture and a drainage bag was attached. It was left in place for 3–5 days postoperatively in the TDT group. In the No-TDT group, no transanal silicone tube drainage was used after the anastomosis. A total of 927 patients (620 in the no-TDT group and 307 in the TDT group) were included in the analysis, and a total of 50 (5.4%) patients were observed to develop AL. After using propensity score matching (PSM) to reduce baseline feature imbalances between the two groups, there were 287 patients in both groups, and the mean retention time of TDT was (4.7 ± 1.2) d. The incidence of AL in the TDT group was significantly lower than that in the no-TDT group (3.8% vs. 8.0%, with a the incidence of AL in the TDT group was significantly lower than that in the non-TDT group (3.8% vs. 8.0%, *P* = 0.034), but the incidence of AL classification was similar (*P* = 0.709). There were no significant differences between the two groups in terms of postoperative complications and postoperative recovery. Multivariate logistic regression analysis revealed that TDT was found to be an independent protective factor for postoperative AL (OR 0.437, 95% CI 0.207–0.923, *P* = 0.030). The elective use of TDT is a simple and effective protective measure for the prevention of AL in patients without stoma after LAR surgery, helping to reduce the probability of AL. This may be a potential alternative DS method for the appropriate population.

## Introduction

Laparoscopy-assisted rectal cancer is increasingly used as an alternative to open surgery for rectal cancer^1,2^. Meanwhile, the double-stapling technique (DST) continues to improve, and an increasing number of patients with rectal cancer have the opportunity to preserve their sphincter^3^. However, the thorny problem of anastomotic leakage (AL) still needs to be addressed. Although many high-quality studies have been devoted to reducing the incidence of AL after laparoscopic-assisted anterior rectal resection (LAR), the incidence remains as high as 6.1–12.3%^3–5^.

Since the first report of the preventive effect of a transanal drainage tube (TDT) on AL in 1997^6^, several trials and meta-analyses have been dedicated to exploring the role of TDT in LAR^4,6–16^. It has been shown that the use of TDT can provide drainage, promote gastrointestinal motility, and reduce postoperative rectal resting pressure in patients compared with those who did not use TDT^17^. In many cases, TDT may be a safe and effective alternative to diverting stomas (DS)^18^. Nevertheless, the conclusions reached in later randomised controlled trials were the opposite^7,8^, and some studies were forced to terminate the study early for ethical reasons^19^. The most recent meta-analyses have reached different conclusions^20,21^. Therefore, the efficacy of TDT for preventing AL after rectal surgery remains unclear.

It is well known that DS repeatedly reduces the probability of AL and the reoperation rate and may even protect patients’ lives^22^. Therefore, even in randomised controlled trials, we cannot randomly choose whether to use DS or not, which causes a certain bias in the studies, and some of them were forced to be terminated early under the pressure of ethics and safety^19^. Meanwhile, clinicians have some subjectivity in the choice of using DS or TDT and may choose DS for a lower anastomotic position and tend to use TDT for a higher position^23^, as well as the small size of the populations included in the studies, which may have led to the inconsistent results of the studies above. Therefore, this study reports a large retrospective study conducted at the Chinese Center for the Diagnosis and Treatment of Colorectal Diseases that excluded patients with DS and used propensity-score matching (PSM) analysis to reduce bias in the selection of patients between the two groups. We aimed to assess whether TDT has a protective effect on AL in postoperative LAR patients without DS, and its impact on postoperative recovery and complications.

Illustration: A 32F silicone tube with more than three lateral holes in the tip was inserted through the anus under laparoscopic guidance, with the tip of the tube extending more than 5 cm beyond the anastomosis. The tube was secured around the anus using skin sutures and a drainage bag was attached. (Drawing by Wang).

## Methods

### Patients and study design

Clinical data of patients with rectal cancer treated at the Zhangzhou Affiliated Hospital of Fujian Medical University between January 2018 and December 2023 were retrospectively collected.

### Inclusion and exclusion criteria

The inclusion criteria: (1) the tumor was ≤ 15 cm from the anus and pathologically diagnosed as primary rectal cancer; (2) the anus was successfully saved, LAR surgery was performed, and end-to-end DST anastomosis was completed, the anastomosis is located 1–10 cm from the rectal dentate line; (3) DS was not performed in the first stage of surgery; (4) complete clinical information.

Exclusion criteria: (1) open surgery; (2) palliative surgery with preoperative combined distant metastases; (3) emergency surgery with preoperative combined acute bowel obstruction or bowel perforation, including failure to pass preoperative colonoscopy; (4) combined with organ resection from other sites; (5) other types of anastomosis-free surgery including abdominal perineal resection, Hartmann’s surgery, and transanal partial resection.

### Treatment

All surgeries are performed by 2 lead surgeons in the same department, both of whom have extensive experience in colorectal surgery and are skilled in this procedure. Standard bowel preparations and oral antibiotics were administered preoperatively to prevent infection^24^. The surgical procedure was performed according to the principles of total mesorectal excision (TME), and all were laparoscopic-assisted^25^. The circular anastomosis was inserted transrectally, and end-to-end DST anastomosis was accomplished endoluminally. Airtightness was routinely assessed using transanal air insufflation^26^.

**Fig. 1:**
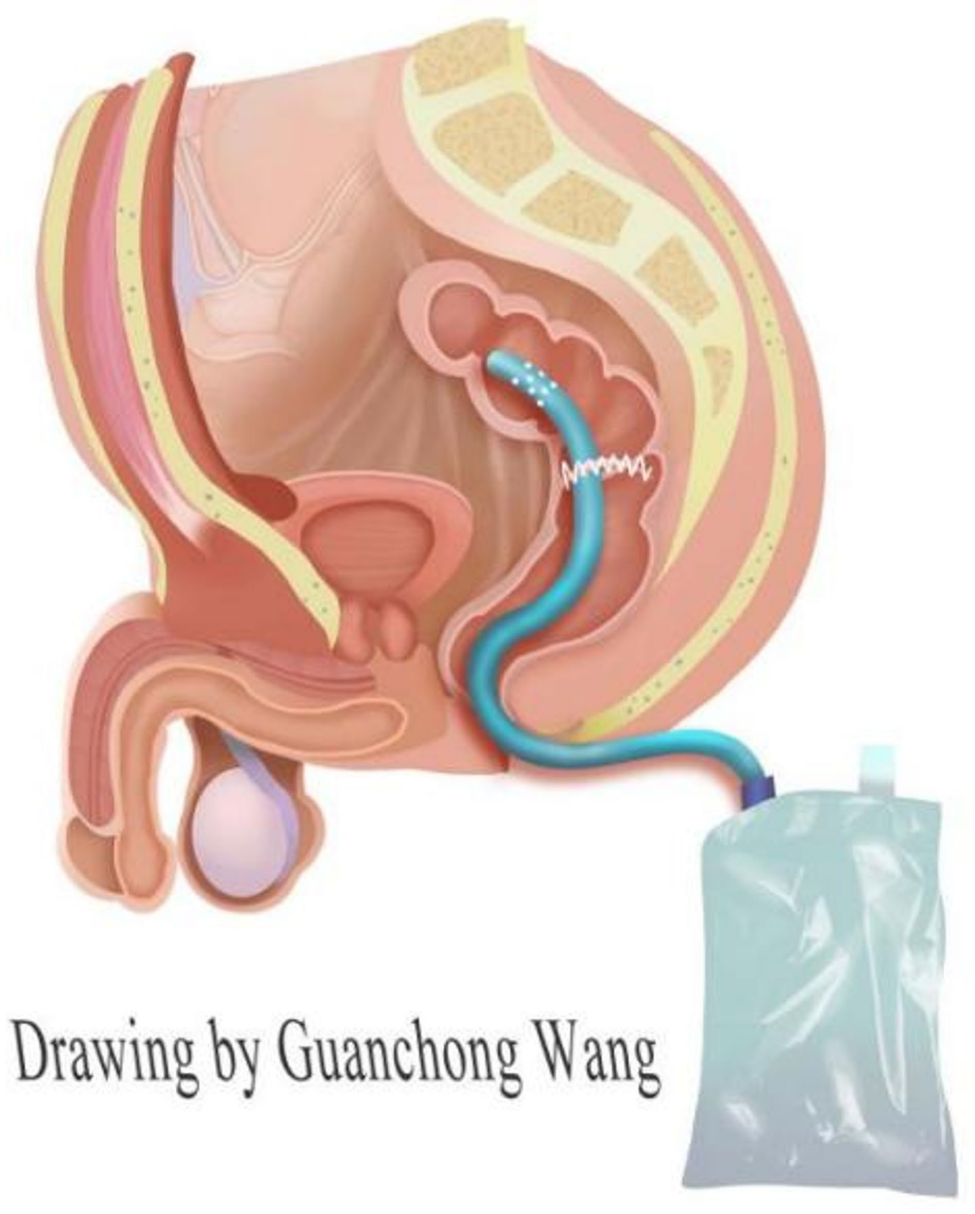
Diagram of the transanal drainage tube.

In the TDT group, after anastomosis of the sigmoid colon to the rectum, a 32F silicone tube was inserted through the anus under laparoscopic-assisted guidance, with its tip exceeding the anastomosis by more than 5 cm above the anastomosis. The tube was secured around the anus using skin sutures and a drainage bag was attached. The TDT group was left in place for 3–5 days postoperatively, unless accidental displacement or intolerance of early extraction occurred, and if AL occurred during this period, the TDT acted as a drain and prolonged the extraction time. If the TDT was removed ≤ 2 days postoperatively was considered to have been removed early or accidentally extubated, and the expected retention goal was not achieved. In the No-TDT group, transanal silicone tube drainage was not used after anastomosis. (Fig. 1).

### Outcomes

The primary endpoint of this study was the occurrence of AL within 30 days after surgery in both groups. The secondary endpoints were anastomotic bleeding, bowel obstruction, bowel recovery, and length of hospital stay.The definition of AL was based on the guidelines issued by the International Rectal Cancer Study Group^27^, and was diagnosed when any of the following conditions were met: (1) abdominal pain, fever, or peritonitis manifested postoperatively, along with a bowel wall defect at the site of the anastomosis, and signs of spillage of the contrast agent on imaging; (2) fecal outflow from pelvic drainage tubes, vagina, or urethra drainage from pelvic drainage tube, vagina, urethra, and palpable anastomotic defect on rectal palpation; (3) intraoperative diagnosis of AL (interruption of bowel wall continuity and defect at the anastomotic site). Grade B is clinically significant and requires active therapeutic intervention but can be improved with conservative treatment.Grade C requires a second open abdominal surgery.

### Statistical analysis

In retrospective observational research, it is an effective and commonly used method to balance the bias of the two groups to achieve an environment similar to that of a randomized controlled study using PSM analysis^28^. This method is well-suited for cohort studies, such as this study, where ethical and risk pressures exist^19^. In this study, matching was performed by 1:1 matching (caliper = 0.2) of the following variables: age, sex, body mass index (BMI), anemia, hypoproteinemia, diabetes, CEA, CA19-9, operation time, intraoperative bleeding, distance to anal verge (DTAV), neoadjuvant chemotherapy, and pT/N/TNM stage.

The characteristics of the two groups were compared before and after PSM. Categorical variables were analyzed using the chi-square test or Fisher’s test, and the rank-sum test was used for grade data. Continuous variables were compared using Student’s t-test (data are expressed as mean ± standard deviation). Logistic regression analysis was used to identify independent predictors of AL. Statistical significance was defined as *P* < 0.05. The above statistical analyses were performed using the SPSS version 26.0 (IBM) software.

## Results

Finally, 927 consecutive patients who met the criteria for consecutive visits were included in the analysis, including 620 in the non-TDT group and 307 in the TDT group. Before PSM, eight patients in the TDT group were dislodged before planned extraction and were still included in the TDT group.

### Patient characteristics

Before PSM, patients in the TDT group had a longer operative time compared to patients in the Non-TDT group (127.3 ± 39.5 vs. 116.8 ± 35.1, *P* < 0.001), and a significantly higher proportion of patients with DTAV 5–10 cm (63.2% vs. 35.0%, *P* < 0.001), which are significantly higher, apparently suggesting a possible relationship between these indicators and postoperative recovery and AL. (Table 1 before PSM).

**Table 1:** Patient characteristics.

Variables	Before PSM			After PSM		
	No-TDT Group (n = 620)	TDT Group (n = 307)	*P*	No-TDT Group (n = 287)	TDT Group (n = 287)	*P*
Sex			0.139			0.860
Male	379 (61.1)	203 (66.1)		190 (66.2)	188 (65.5)	
Female	241 (38.9)	104 (33.9)		97 (33.8)	99 (34.5)	
Age (years)	63.6 ± 10.8	64.0 ± 10.8	0.556	63.9 ± 11.1	63.9 ± 11.0	0.982
BMI (kg/m^2^)	22.6 ± 3.5	22.8 ± 3.3	0.441	22.8 ± 3.4	22.8 ± 3.3	0.944
Anemic			1.000			0.752
No	610 (98.4)	302 (98.4)		281 (97.9)	283 (98.6)	
Yes	10 (1.6)	5 (1.6)		6 (2.1)	4 (1.4)	
Hypoproteinemia			0.425			1.000
No	593 (95.6)	290 (94.5)		272 (94.8)	272 (94.8)	
Yes	27 (4.4)	17 (5.5)		15 (5.2)	15 (5.2)	
Diabetes			0.754			0.459
No	520 (83.9)	255 (83.1)		228 (79.4)	235 (81.9)	
Yes	100 (16.1)	52 (16.9)		59 (20.6)	52 (18.1)	
CEA (ng/ml)			0.589			0.798
< 5	389 (62.7)	187 (60.9)		176 (61.3)	173 (60.3)	
≥ 5	231 (37.3)	120 (39.1)		111 (38.7)	114 (39.7)	
CA19-9 (U/ml)			0.124			0.495
< 37	542 (87.4)	257 (83.7)		238 (82.9)	244 (85.0)	
≥ 37	78 (12.6)	50 (16.3)		49 (17.1)	43 (15.0)	
Operation time (min)	116.8 ± 35.1	127.3 ± 39.5	< 0.001	123.6 ± 39.0	125.5 ± 37.2	0.550
Bleeding (ml)	63.4 ± 40.1	65.0 ± 41.0	0.587	65.5 ± 40.9	64.9 ± 41.6	0.861
DTAV (cm)			< 0.001			0.352
≤ 5	39 (6.3)	12 (3.9)		6 (2.1)	12 (4.2)	
5–10	217 (35.0)	194 (63.2)		178 (62.0)	176 (61.3)	
≥ 10	364 (58.7)	101 (32.9)		103 (35.9)	99 (34.5)	
Neoadjuvant chemotherapy			0.864			0.779
No	605 (97.6)	299 (97.4)		281 (97.9)	280 (97.6)	
Yes	15 (2.4)	8 (2.6)		6 (2.1)	7 (2.4)	
pT stage			0.860			0.969
T0–1	53 (8.5)	26 (8.5)		25 (8.7)	25 (8.7)	
T2	82 (13.2)	47 (15.3)		38 (13.2)	42 (14.6)	
T3	272 (43.9)	132 (43.0)		127 (44.3)	126 (43.9)	
T4	213 (34.4)	102 (33.2)		97 (33.8)	94 (32.8)	
pN stage			0.719			0.551
N0	345 (55.6)	167 (54.4)		147 (51.2)	155 (54.0)	
N +	275 (44.4)	140 (45.6)		140 (48.8)	132 (46.0)	
pTNM stage			0.850			0.697
0	11 (1.8)	5 (1.6)		4 (1.4)	5 (1.7)	
I	98 (15.8)	59 (19.2)		45 (15.7)	53 (18.5)	
II	236 (38.1)	103 (33.6)		98 (34.1)	97 (33.8)	
III	275 (44.4)	140 (45.6)		140 (48.8)	132 (46.0)	

PSM, propensity-score matched; TDT, transanal drainage tube; BMI, body mass index; CEA, circumferential resection margin; CA19-9, carbohydrate antigen 19–9; DTAV, distance to the anal verge.

After PSM, a new cohort was obtained, both groups contained 287 patients, including operative time. DTAV indicators were well-matched, and the basic patient-specific differences were not statistically significant (all *P* > 0.05), specifically higher comparability. 8 patients (2.8%) in the TDT group did not complete the TDT because they could not tolerate the early removal of the TDT. (Table 1, after PSM).

### Primary endpoint

Before PSM, 50 cases of postoperative AL were observed in this cohort, with an overall incidence of 5.4%, including 26 grade C AL leading to reoperation and 24 grade A + B AL. The differences in postoperative AL and AL grades were not statistically significant.

After PSM, the average preservation time of TDT was 4.7 ± 1.2 days. In terms of the probability of AL, the TDT group had a significantly lower incidence than the non-TDT group (3.8% vs. 8.0%), and the difference was statistically significant (*P* = 0.034). However, the incidence of grade C AL was similar in terms of the AL grade (54.5% vs. 65.2%, *P* = 0.709) (Table 2).

**Table 2:** Comparison of postoperative outcomes between the two groups of patients.

Variables	Before PSM			After PSM		
	No-TDT group	TDT group	*P*	No-TDT group	TDT group	*P*
Time of TDT (days)	No value	4.8 ± 1.2	No value	No value	4.7 ± 1.2	No value
Anastomotic leakage			0.159			0.034
No	582 (93.9)	295 (96.2)		264 (92.0)	276 (96.2)	
Yes	38 (6.1)	12 (3.9)		23 (8.0)	11 (3.8)	
Anastomotic leakage Grade			0.874			0.709
Grade B	18 (47.4)	6 (50.0)		8 (34.8)	5 (45.5)	
Grade C	20 (52.6)	6 (50.0)		15 (65.2)	6 (54.5)	
Anastomotic bleeding			0.182			0.279
No	610 (98.4)	298 (97.1)		282 (98.3)	278 (96.9)	
Yes	10 (1.6)	9 (2.9)		5 (1.7)	9 (3.1)	
Intestinal obstruction			0.182			1.000
No	599 (96.6)	298 (97.1)		279 (97.2)	279 (97.2)	
Yes	21 (3.4)	9 (2.9)		8 (2.8)	8 (2.8)	
Time of anal gas evacuation (days)	2.4 ± 1.7	2.4 ± 1.0	0.981	2.4 ± 1.6	2.4 ± 1.0	0.488
Time of return to regular diet (days)	3.7 ± 1.9	3.7 ± 1.2	0.912	3.7 ± 1.8	3.7 ± 1.2	0.435
Length of postoperative hospitalization (days)	9.1 ± 5.1	9.3 ± 4.0	0.368	9.5 ± 6.2	9.3 ± 4.0	0.333

TDT, transanal drainage tube.

PSM, propensity-score matched; TDT, transanal drainage tube; BMI, body mass index; CEA, carcinoembryonic antigen; CA19-9, carbohydrate antigen 19–9; DTAV, distance to the anal verge.

### Secondary endpoints

We further explored whether TDT had any effect on other postoperative complications or on postoperative recovery. There were no statistically significant differences in the probability of anastomotic bleeding, intestinal obstruction, time to anal evacuation/resumption of a normal diet, and postoperative hospital stay, either before or after PSM (*P* > 0.05) (Table 2).

### Independent impact factors of AL

We further explored the independent impact factors of AL using logistic regression modeling. Before PSM, multivariate regression analysis showed that, carcinoembryonic antigen (CEA) level and operative time had an independent effect on postoperative AL. However, TDT did not have a protective effect on postoperative AL (odds ratio [OR] 0.519, 95% CI 0.259–1.037, *P* = 0.063) (Table 3).

**Table 3:** Univariate and multivariate logistic regression analysis of rectal anastomotic leakage before PSM (n = 927).

Variables	Univariate analysis		Multivariate analysis	
	OR (CI 95%)	*P* Value	OR (CI 95%)	*P* Value
TDT (yes vs. no)	0.519 (0.259–1.037)	0.063		
Sex (Male vs. Female)	0.711 (0.367–1.376)	0.311		
Age (years)	0.969 (0.942–0.996)	0.027		
BMI (kg/m^2^)	0.953 (0.870–1.043)	0.296		
Anemic (yes vs. no)	0.962 (0.109–8.305)	0.965		
Hypoproteinemia (yes vs. no)	1.205 (0.312–4.656)	0.787		
CEA (ng/ml)	2.107 (1.113–3.990)	0.022	2.286 (1.276–4.093)	0.005
CA19-9 (U/ml)	1.370 (0.636–2.953)	0.422		
Diabetes (yes vs. no)	1.373 (0.636–2.964)	0.420		
Operation time (min)	1.011 (1.004–1.018)	0.001	1.010 (1.004–1.016)	0.001
DTAV (≥ 10 vs. 5–10, ≤ 5 cm)	0.826 (0.512–1.333)	0.434		
Intraoperative bleeding (ml)	0.999 (0.993–1.006)	0.884		
Neoadjuvant chemotherapy (yes vs. no)	1.476 (0.321–6.790)	0.617		
pT stage (T4 vs. T0–3)	1.667 (0.883–3.149)	0.115		
pN stage (N + vs. N0)	0.520 (0.157–1.724)	0.285		
pTNM stage (III vs. II–0)	1.148 (0.490–2.693)	0.75		

PSM, propensity-score matched; TDT, transanal drainage tube; BMI, body mass index; CEA, carcinoembryonic antigen; CA19-9, carbohydrate antigen 19–9; DTAV, distance to the anal verge.

As described previously, to reduce retrospective study selection bias, we balanced the unbalanced factors between the 2 groups of patients using the PSM method to improve comparability. After PSM, we explored the independent influence of AL. The results showed that CEA level (OR 2.327, 95% CI 1.139–4.754, *P* = 0.020) and operation time (OR 1.010, 95% CI 1.003–1.017, *P* = 0.007) still had independent influences on postoperative AL. TDT was found to be an independent protective factor for postoperative AL (OR 0.437, 95% CI 0.207–0.923, *P* = 0.030) (Table 4).

**Table 4:** Univariate and multivariate logistic regression analysis of rectal anastomotic leakage after PSM (n = 574).

Variables	Univariate analysis		Multivariate analysis	
	OR (CI 95%)	*P* Value	OR (CI 95%)	*P* Value
TDT (yes vs. no)	0.444 (0.206–0.958)	0.039	0.437 (0.207–0.923)	0.030
Sex (Male vs. Female)	0.510 (0.208–1.252)	0.142		
Age (years)	0.966 (0.933–0.999)	0.045		
BMI (kg/m^2^)	0.899 (0.794–1.017)	0.092		
Anemic (yes vs. no)	1.158 (0.118–11.381)	0.900		
Hypoproteinemia (yes vs. no)	1.418 (0.343–5.864)	0.630		
CEA (ng/ml)	1.816 (0.800–4.118)	0.153	2.327 (1.139–4.754)	0.020
CA19-9 (U/ml)	1.334 (0.535–3.331)	0.537		
Diabetes (yes vs. no)	2.075 (0.879–4.900)	0.096		
Operation time (min)	1.011 (1.003–1.018)	0.007	1.010 (1.003–1.017)	0.007
DTAV (≥ 10 vs. 5–10, ≤ 5 cm)	1.021 (0.514–2.026)	0.953		
Intraoperative bleeding (ml)	0.997 (0.987–1.006)	0.467		
Neoadjuvant chemotherapy (yes vs. no)	1.233 (0.135–11.293)	0.853		
pT stage (T4 vs. T0–3)	1.443 (0.656–3.176)	0.362		
pN stage (N + vs. N0)	0.867 (0.189–13.983)	0.854		
pTNM stage (III vs. II–0)	0.987 (0.346–2.817)	0.980		

PSM, propensity-score matched; TDT, transanal drainage tube; BMI, body mass index; CEA, carcinoembryonic antigen; CA19-9, carbohydrate antigen 19–9; DTAV, distance to the anal verge.

## Discussion

This study included 927 patients with rectal cancer without DS to evaluate the effect of TDT on AL after LAR. After PSM 1:1 matching, the results showed that the proportion of AL in the TDT group was significantly lower than that of patients in the Non-TDT group, and the difference was statistically significant (3.8% vs. 8.0%, *P* = 0.034). Meanwhile, TDT was confirmed to be an independent protective factor for AL by multifactorial regression (OR 0.437, 95% CI 0.207–0.923, *P* = 0.030), but failed to reduce the rate of postoperative grade C AL.

AL is a frequent and serious complication of rectal sphincter preservation surgery, resulting in a huge economic burden and severely threatening the life of the patient^3,29^. Since TDT can elicit the rectal contents in a timely manner, thereby reducing the resting pressure in the intestinal lumen, theoretically, it has a protective effect on postoperative anastomosis, but it still cannot completely avoid postoperative AL as well as the serious complications that it may cause^6,17^. Several previous RCTs have aimed to confirm the efficacy of TDT, but the conclusions drawn remain inconsistent. Xiao et al. prospectively included 439 patients with a DTAV ≤ 15 cm, excluding neoadjuvant chemotherapy and DS patients. Patients were likely to use handsewn or double-staple techniques for anastomosis, but did not disclose that the TDT group used the caliber of silicone tubing; ultimately, AL was considered significantly lower in TDT patients (4.0% vs. 9.6%, *P* = 0.026)^17^. Tamura et al. in their prospective study included a relatively small sample size; only 161 patients with DTAV ≤ 15 cm were enrolled, and the TDT group used 20-24F silicone tubing with a smaller caliber and poorer drainage, with no difference in the AL rate between the two groups (10.1% vs. 14.1%, *P* = 0.445)^8^. Unlike the previous two RCT studies, Zhao et al. included 560 patients with DTAV ≤ 10 cm and also excluded preoperative neoadjuvant chemotherapy, the decision of whether to do DS in the study was made by the surgeon after the anastomosis, and 28F silicone tubing was used in the TDT group. Further subgroup analyses were done according to the presence or absence of DS, taking into account the possible effects of DS, but ultimately, it was concluded that TDT had no protective effect (OR 0.95, 95% CI 0.51–1.77, *P* = 0.87)^7^. In addition to the aforementioned reasons, such as inconsistent TDT retention times and different surgical approaches, including laparoscopic or open surgery, a combination of reasons may lead to inconsistent conclusions from different RCT studies.

Several newly published high-quality meta-analyses have shown a protective effect of TDT, especially among patients without DS, similar to the findings of the present study^13,14^. In a meta-analysis, Shiki et al. concluded that TDT reduced the incidence of AL in a subgroup of 955 patients without DS (OR 0.50, 95% CI 0.29–0.86, *P* = 0.012), but concluded that it was not protective if DS patients were included in the whole population^14^. This is also supported by another larger meta-analysis, which suggests that patients without DS may be the population with the greatest benefit from TDT^13^. Furthermore, a meta-analysis concluded that TDT has a greater role in reducing the chance of reoperation after the occurrence of AL, that is, preventing grade C AL, which brings certain clinical benefits^15,16^.

Unfortunately, no study has clearly shown the indications for its use in LAR. However, the use of TDT may influence the surgeon’s decision on the application of DS, which has been suggested to be applied in patients with high expected reoperation rates or mortality, and TDT has been considered a potential alternative to DS^30^. Although PSM was performed in this study to reduce confounders between the 2 groups, we have to recognise that there is still some selection bias when surgeons are choosing whether or not to use TDT. For example, in the non-DS population, patients with a DTAV of 5–10 cm were more likely to be selected for TDT (63.2% vs. 36.8%, *P* < 0.001) as patients with a DTAV of ≤ 5 cm required DS or even APR, whereas patients with a DTAV of ≥ 10 cm had a relatively low incidence of AL for which TDT was less commonly used. Alternatively, patients with longer operative times are more likely to be treated with TDT.In short, intraoperative use of TDT requires a simple and feasible method of anastomotic protection chosen by the surgeon on the basis of comprehensive post-anastomotic judgement when the anastomosis is deemed to be less than optimal and at the expense of not wanting to perform a DS. Finally, we advocate that silicone tubing used for TDT should be recommended to choose larger calibre silicone tubing, such as 32F, with multiple side holes at the tip to ensure drainage^31^.

This study had several limitations. First, as a retrospective study, although the sample size was sufficiently large, issues such as confounders and selection bias unavoidably existed; therefore, we utilized the PSM method to minimize between-group bias. Second, we excluded patients with incomplete information where the incidence of AL might have been overestimated or underestimated. Third, this study only included patients who underwent laparoscopic-assisted surgery, and further exploration is needed in patients who undergo open surgery. Finally, the depth of TDT placement may not be exactly the same in different patients, but it can be ensured to be > 5 cm above the anastomosis, and factors such as optimal TDT placement depth and placement time need to be further explored. However, to the best of our knowledge, in terms of sample size and exclusion of DS bias, our study, either before or after PSM, is the largest retrospective controlled study to explore the efficacy of TDT for the prevention of postoperative AL after LAR to date.

## Conclusion

The elective use of TDT is a simple and effective protective measure for the prevention of AL in patients without a stoma after laparoscopic AR surgery, helping to reduce the probability of AL. It may be a potential alternative DS method among the appropriate population, but more studies are needed to clarify the indications for its clinical use.

## Data Availability

All data obtained or analyzed during this study are included in the article. Data analyzed during the study can be obtained from the corresponding author if necessary.
